# Investigation of Overlapped Twisted Tapes Inserted in a Double-Pipe Heat Exchanger Using Two-Phase Nanofluid

**DOI:** 10.3390/nano10091656

**Published:** 2020-08-24

**Authors:** Mehdi Ghalambaz, Hossein Arasteh, Ramin Mashayekhi, Amir Keshmiri, Pouyan Talebizadehsardari, Wahiba Yaïci

**Affiliations:** 1 Institute of Research and Development, Duy Tan University, Da Nang 550000, Vietnam; mehdi.ghalambaz@duytan.edu.vn; 2 Faculty of Electrical–Electronic Engineering, Duy Tan University, Da Nang 550000, Vietnam; 3 Department of Mechanical Engineering, Isfahan University of Technology, Isfahan 8415683111, Iran; h.arasteh.iut@gmail.com; 4 Research & Development Team, Couette Limited, Altrincham WA14 2PX, UK; info@couette.co.uk; 5 Department of Mechanical, Aerospace and Civil Engineering (MACE), The University of Manchester, Manchester M13 9PL, UK; A.Keshmiri@manchester.ac.uk; 6 Metamaterials for Mechanical, Biomechanical and Multiphysical Applications Research Group, Ton Duc Thang University, Ho Chi Minh City 758307, Vietnam; 7 Faculty of Applied Sciences, Ton Duc Thang University, Ho Chi Minh City 758307, Vietnam; 8 CanmetENERGY Research Centre, Natural Resources Canada, 1 Haanel Drive, Ottawa, ON K1A 1M1, Canada

**Keywords:** overlapped twisted tape, two-phase, heat transfer, nanofluid, double-pipe

## Abstract

This study investigated the laminar convective heat transfer and fluid flow of Al_2_O_3_ nanofluid in a counter flow double-pipe heat exchanger equipped with overlapped twisted tape inserts in both inner and outer tubes. Two models of the same (co-swirling twisted tapes) and opposite (counter-swirling twisted tapes) angular directions for the stationary twisted tapes were considered. The computational fluid dynamic simulations were conducted through varying the design parameters, including the angular direction of twisted tape inserts, nanofluid volume concentration, and Reynolds number. It was found that inserting the overlapped twisted tapes in the heat exchanger significantly increases the thermal performance as well as the friction factor compared with the plain heat exchanger. The results indicate that models of co-swirling twisted tapes and counter-swirling twisted tapes increase the average Nusselt number by almost 35.2–66.2% and 42.1–68.7% over the Reynolds number ranging 250–1000, respectively. To assess the interplay between heat transfer enhancement and pressure loss penalty, the dimensionless number of performance evaluation criterion was calculated for all the captured configurations. Ultimately, the highest value of performance evaluation criterion is equal to 1.40 and 1.26 at inner and outer tubes at the Reynolds number of 1000 and the volume fraction of 3% in the case of counter-swirling twisted tapes model.

## 1. Introduction

In thermal engineering systems, convective heat transfer involves wide applications, including fuel cells [1], refrigeration [2], electronic device cooling [3], solar air collectors [4], and aerospace engineering [5]. The rise of energy demand at the turn of the new century has provoked the attention of researchers in this field of study to promote the efficiency of thermal energy systems. To increase the thermohydraulic performance of engineering systems, different techniques have been proposed, among which using twisted tape inserts has been widely used [6,7]. Twisted tape generates a swirl flow that disturbs boundary layers of velocity and temperature inside the tube, which directly improves the heat transfer coefficient in a tube [8,9,10,11]. Applying additive nanoparticles to the base fluid to enhance the thermophysical properties of the working fluid has also been considered as an effective technique to modify heat transfer [12,13]. The hybrid nanofluids such as Ag–MgO–water [14,15], Ag–TiO_2_ [16], Cu–Al_2_O_3_ [16,17], and MgO–MWCNTs/EG [18] as well as using of nonencapsulated phase change material suspension [19] are also promising for enhancement of heat transfer. On the other hand, combining different techniques to promote the thermohydraulic performance of thermal engineering systems as much as possible has been paid a lot of attention in recent years [20,21].

One of the recent compound methods in heat transfer enhancement is the hybrid employment of nanofluids and inserted twisted tapes [22]. In what follows, the recent achievements of such problem are discussed. Some researchers have focused on the effects of pitch and width ratios of twisted tapes. Jaramillo et al. [23] used the twisted tape inserts in a parabolic collector. Their results indicate the higher thermal performance of the collector in low twisted ratios operating at low values of Reynolds number. This type of collector equipped with wall-detached twisted tape inserts was also investigated numerically by Mwesigye et al. [24]. They showed that, as twist ratio increases and width ratio reduces, the optimal Reynolds number enhances. A numerical study of a tri-lobbed tube equipped with twisted tapes was performed by Esfe et al. [25]. They showed that pitch ratio enhancement causes an increase in Nusselt number, friction factor, and overall thermal performance.

Using twisted tapes in double-pipe heat exchangers has also been examined by many researchers. The use of twisted tape elements in the inner tube of a double-pipe heat exchanger with clockwise and counterclockwise swirling directions was performed experimentally by Man et al. [26]. The results indicate a higher heat exchanger efficiency by using twisted tape inserts so that the highest PEC number was equal to 1.42 and was reported under the empirical conditions. The use of twisted tapes in a double-tubes at various pitch ratios was examined by Lim et al. [27]. They predicted heat transfer characteristics for different Reynolds number values. Ravi Kumar et al. [28] evaluated the thermal performance of a double-tube U-bend system inserting twisted tapes using the ε-NTU method and found higher effectiveness and number of heat transfer units.

Another enhancement technique of twisted tape inserts is to enhance the flow mixing and secondary flow effects by changing the geometry of twisted tapes. Saylroy and Eiamsa-ard [29] analyzed square-cut twisted-tape inserts in a circular tube and showed the highest PEC number of 1.37 with thermal performance improvement of about 1.32 times higher than the classically twisted tape inserts. In another study [30], they investigated the multi-channel twisted tapes with constant wall temperature. They showed an enhancement of laminar convection heat transfer by applying such twisted tapes. He et al. [31] applied to cross hollow twisted tape inserts in a tube to study the thermal characteristics empirically. They showed that the PEC number ranges from 0.87 to 0.98 for Reynolds numbers varying from 5600 to 18,000. Samruaisin et al. [32] embedded quadruple twisted tapes in a tube to analyze experimentally and numerically the efficacies of twisted tape arrangement and free space ratio in a turbulent regime. They reported the maximum value of 1.27 for PEC numbers in the range of studied operational conditions. Ruengpayungsak et al. [33] investigated centrally perforated twisted tape inserts numerically in both laminar and turbulent regimes. They reported the maximum value for PEC number equal to almost 8.92 and 1.33 for laminar and turbulent regimes, respectively. Hasanpour et al. [34] investigated a corrugated tube inserting twisted tapes using the optimization method on experimental tests. The maximum heat transfer rate is gained at V-cut twisted tape model, and the minimum pressure drop is achieved at perforated twisted tape model.

The application of using nanofluid combined with twisted tape is also studied. Sunder et al. [26] examined a solar water heater experimentally using passive techniques of nanofluid and twisted tape. They concluded that, for Reynolds number equal to 13,000 with 3% nanoparticles, 49.75% enhancement in heat transfer was achieved for the case in the presence of twisted tape with a pitch to diameter ratio of 5, while it is equal to only 21% for the plain tube. Qi et al. [35] accomplished an experimental study of convective nanofluid employing rotating and static built-in twisted tapes. They showed that using rotating twisted tape along with the nanofluid results in a 101.6% enhancement in heat transfer.

Another form of twisted tape inserts technique to enhance the thermal performance over the pumping power penalty, namely overlapped twisted tapes. Hong et al. [36] conducted an empirical study of a spiral grooved tube equipped with twin overlapped twisted tapes with Reynolds numbers of 8000 to 22,000, showing an enhancement in both heat transfer and friction coefficient as the overlapped twisted ratio augments. In another study [36], they employed overlapped multiple twisted tapes in a similar experiment. They showed higher entropy generation due to friction resistance and lower entropy generation due to heat transfer for a higher number of tapes and lower overlapped twisted ratios. Eiamsa-Ard and Samravysin [37] conducted an empirical study on overlapped-quadruple twisted tape inserts comparing to typical quadruple twisted tape elements for various Reynolds number under a turbulent regime. They reported the maximum value of 1.58 for PEC number at Reynolds number of 5000. Eiamsa-Ard et al. [37] in a numerical and experimental study employed overlapped dual twisted tapes along with nanofluid using TiO_2_ nanoparticles. They showed an 89% enhancement in heat transfer and 113% in thermal performance compared with the plain tube. Overlapped dual twisted tapes along with Al_2_O_3_ nanofluid were studied by Rudrabhiramu et al. [38]. They reported that using 1% nanofluid volume concentration and twisted tape twist pitch ratio of 2 causes the best result compared to all other captured cases.

According to the aforementioned literature review, the simultaneous implementation of various enhancement techniques leads to hybrid effects that offer higher thermal performance relative to the corresponding values achieved from each method. Applying both nanofluid and twisted tape inserts has been introduced as a promising way. However, reviewing the preceding papers reveals that the effects of overlapped twisted tape inserts in inner and outer tubes of double-pipe heat exchangers are rarely discussed in the literature. Motivated by this research gap, the main objective of this study was to investigate the heat transfer enhancement and fluid flow characteristics of Al_2_O_3_ nanofluid in a double-pipe counter flow heat exchanger equipped with overlapped twisted tape inserts in inner and outer tubes with the same and opposite angular directions. Different effective parameters, including Reynolds number and the volume fraction of nanoparticles, were also analyzed by various contour plots and diagrams.

## 2. Problem Statement

**Figure 1:**
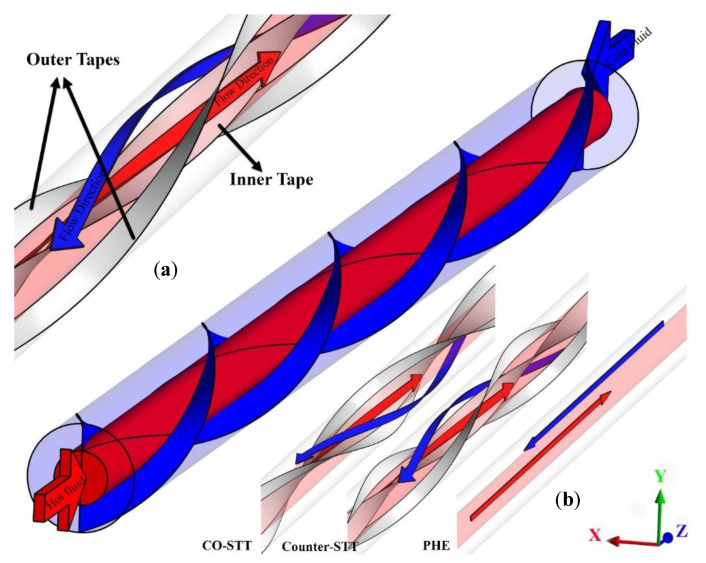
(**a**) Current study Schematic diagram with overlapped twisted tapes; and (**b**) comparison of the different proposed cases.

Figure 1 schematically shows the counter flow double-pipe system equipped with overlapped twisted tapes in inner and outer tubes. The inner and outer tubes diameters are 10 and 29 mm, the thickness of the twisted tape is 0.4 mm with equivalent pitches of 100 mm. Two models for embedding the overlapped twisted tapes are considered: in the first model, the inner and outer twisted tapes swirl in the same angular direction (Co-STT as an abbreviation of co-swirling twisted tapes), and, in the second model, the inner and outer twisted tapes swirl in an opposite angular direction (Counter-STT as an abbreviation of counter-swirling twisted tapes). The plain heat exchanger (PHE) is also studied compared with the twisted tape cases. Al_2_O_3_–water nanofluid enters the inner and outer tubes at a temperature of 300 K, considering four different Reynolds numbers.

### 2.1. Governing Equations

A steady-state laminar incompressible Al_2_O_3_–water nanofluid flow neglecting the effects of radiation and viscosity losses is studied. The two-phase mixture model is used to simulate Al_2_O_3_ nanoparticles dispersed in water as follows:

- Continuity equation:
  (1) $$\vec{\nabla }.\left(\rho_{m}{\vec{V}}_{m}\right)=0$$
- Momentum equation:
  (2) $$\vec{\nabla }.\left(\rho_{m}{\vec{V}}_{m}\;{\vec{V}}_{m}\right)=-\vec{\nabla }p+\nabla .\left[\mu_{m}\left(\vec{\nabla }\;{\vec{V}}_{m}+\vec{\nabla }\;{\vec{V}}_{m}{}^{T}\right)\right]+\rho_{m}g+\vec{F}-\vec{\nabla }.\left(\sum_{k=1}^{n}\phi_{k}\rho_{k}{\vec{V}}_{dr,k}{\vec{V}}_{dr,k}\right)$$
  where the drift velocity of the secondary phase drift is computed from:
  (3) $${\vec{V}}_{dr,k}={\vec{V}}_{k}-{\vec{V}}_{m}$$
- Energy equation:
  (4) $$\vec{\nabla }.\left[\sum_{k=1}^{n}\left(\rho_{k}c_{pk}\right)\phi_{k}{\vec{V}}_{k}T\right]=\vec{\nabla }.k_{m}\vec{\nabla }T$$

$V_{m}$ and $\rho_{m}$ are the average mass velocity and mixture density, respectively, which are determined as:

(5) $${\vec{V}}_{m}=1/\rho_{m}\left(\sum_{k=1}^{n}\phi_{k}\rho_{k}{\vec{V}}_{k}\right)$$

(6) $$\rho_{m}=\sum_{k=1}^{n}\phi_{k}\rho_{k}$$

In Equation (5), $\phi_{k}$ represents the volume fraction of the mixture *k*th phase.

The relative velocity for the two phases and the mixture viscosity are calculated as:

(7) $$\vec{V_{pf}}=\vec{V_{p}}-\vec{V_{f}}$$

(8) $$\mu_{m}=\sum_{k=1}^{n}\phi_{k}\mu_{k}$$

The relative velocity proposed by Manin [39] and Shiller Newman drag function [40] are used as follows:

(9) $${\vec{V}}_{pf}=\frac{\rho_{p}d_{p}^{2}\left(\rho_{p}-\rho_{m}\right)}{18\mu_{f}f_{drag}\rho_{p}}\left[g-\left(\vec{\nabla }.{\vec{V}}_{m}\right){\vec{V}}_{m}\right]$$

(10) $$f_{drag}=\{\begin{array}{c} 1+0.15Re_{p}^{0.687}\;;\;Re_{p}\leq 1000\\ 0.0183Re_{p}\;\;\;;\;Re_{p}>1000 \end{array}$$

Hence, the drift velocity is given as:

(11) $${\vec{V}}_{dr,p}={\vec{V}}_{pf}-\sum_{k=1}^{n}\left(\frac{\phi_{k}\rho_{k}}{\rho_{m}}{\vec{V}}_{fk}\right)$$

### 2.2. Nanofluid Thermo-Physical Properties

The thermophysical properties of water–Al_2_O_3_ nanofluid are presented in Equations (12)–(15).

The density of water–Al_2_O_3_ nanofluid is calculated using Khanafer and Vafai [41] model as follows:

(12) $$\rho_{nf}=\left(1-\phi \right)\rho_{f}+\phi \rho_{s}$$

The specific heat capacity of water–Al_2_O_3_ nanofluid is defined using Bianco et al. [42] model as Equation (13):

(13) $${\left(\rho c_{p}\right)}_{nf}=\left(1-\phi \right){\left(\rho c_{p}\right)}_{f}+\phi {\left(\rho c_{p}\right)}_{s}$$

The effective dynamic viscosity of water–Al_2_O_3_ nanofluid is determined using Maiga et al. [43] model as below:

(14) $$\mu_{nf}=\frac{\mu_{f}}{{\left(1-\phi \right)}^{2/5}}$$

The effective conductivity of water–Al_2_O_3_ nanofluid is calculated using Qi et al. [35] model as the following equation:

(15) $$k_{nf}=1+2.72\phi +4.97\phi^{2}$$

The water–Al_2_O_3_ nanofluid with three values of volume fractions are listed in Table 1.

### 2.3. Boundary Conditions and Data Deduction

At the heat exchanger, inner and outer tubes inlet, uniform velocity and temperature profiles are applied to the boundary conditions. At the outlets, zero relative gauge pressure is considered. The outer wall of the outer tube is thermally isolated, and the coupled-wall boundary is used in the interface wall.

The hydrothermal parameters employed in this study are defined as follows:

(16) $$D_{h}=\frac{4A}{P}$$

(17) $$f=\frac{2\Delta PD_{h}}{\rho_{nf}U_{m}^{2}L}$$

(18) $$h=\frac{q}{T_{w}-T_{b}}$$

(19) $$Nu_{x}=\frac{h_{x}D_{h}}{k}$$

(20) $$Nu_{avg}=\frac{1}{L}\overset{L}{\underset{0}{\int }}Nu_{x}dx$$

(21) $$PEC=\frac{Nu/Nu_{0}}{{\left(f/f_{0}\right)}^{1/3}}$$

## 3. Numerical Procedure

The commercial ANSYS-FLUENT code was employed in this study using the Coupled algorithm for velocity–pressure coupling using the second-order upwind scheme. The convergence criteria for energy and Navier–Stokes equations were considered 10^−6^.

### 3.1. Grid Study

**Figure 2:**
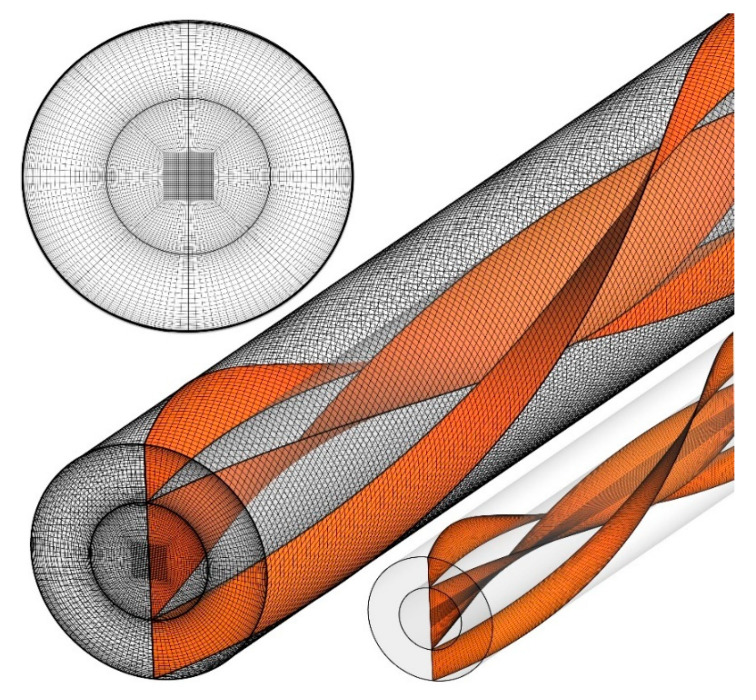
The meshing of the computational domain.

Figure 2 depicts the meshing of the computational domain, which is fully structured to enhance the quality of the results along with reducing the computational time. Finer mesh near the walls due to the presence of severe velocity and temperature gradients is also considered.

Grids with different numbers of mesh elements are examined considering the average Nusselt number as the criteria to examine the grid independency. Table 2 presents the cases considered for mesh independence analysis. The percentage of the difference between two consecutive grids are also presented. Case 5 is chosen for all the simulations, since using finer meshing leads to relative errors less than two percent.

### 3.2. Validation

To verify the code, the experimental results of Qi et al. [35] for the average Nusselt number for laminar nanofluid flow in a circular tube made of stainless steel using stationary and rotating twisted tapes were used. They used a motor to drive the rotation of the twisted tape which was set to 5 RPM. They experimentally examined different concentrations of TiO_2_/water nanofluid (0.1%, 0.3%, and 0.5%) at different Reynolds numbers (600–7000) in a circular tube, with inner diameter, thickness and length of 22, 2, and 1400 mm (only the middle section (1000 mm) was used as the test section), respectively, equipped with twisted tape inserts with the length, pitch size, width, and thickness of 1600, 100, 16, and 2 mm, respectively. They chose relatively low mass concentrations of nanoparticles to reach a better stability for the nanofluid. They discussed the results based on the line relationship between the shear stress and shear rate that TiO_2_ nanofluids can be approximately regarded as a kind of Newtonian fluid, and the non-Newtonian effects can be ignored.

**Figure 3:**
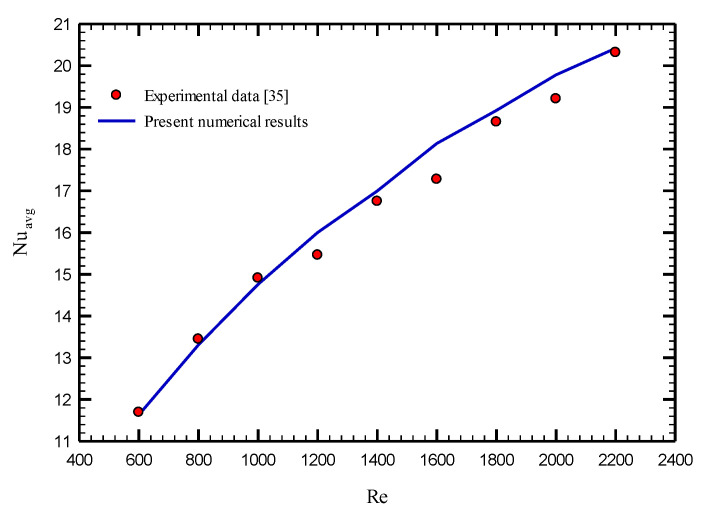
Validation results with the study of Qi et al. [35]. Reproduced with permission from [35]. Copyright Elsevier, 2020.

Figure 3 displays the average Nusselt number for the stationary twisted tape with nanofluid volume concentration of 0.5% at different Reynolds numbers ranging from 600 to 2200. As shown, the results are in excellent agreement with the experimental data of Qi et al. [35] where the maximum difference is less than 3.5%, as displayed in Table 3.

## 4. Results

Two models for embedding the overlapped twisted tapes were considered: in the first model, the inner and outer twisted tapes swirl in the same angular direction (Co-STT), and, in the second, model, the inner and outer twisted tapes swirl in an opposite angular direction (Counter-STT). The proposed models were analyzed for four Reynolds numbers (250, 500, 750, and 1000) employing Al_2_O_3_ nanofluid at four concentrations (0, 1, 2, and 3%). It should be noted that, in the following, first, the different cases shown in Figure 1 are examined using pure water as the HTF to find the best configuration. Then, for the best system, the effects of nanoparticle concentration are assessed.

### 4.1. Effect of Double-Pipe Configurations

**Figure 4:**
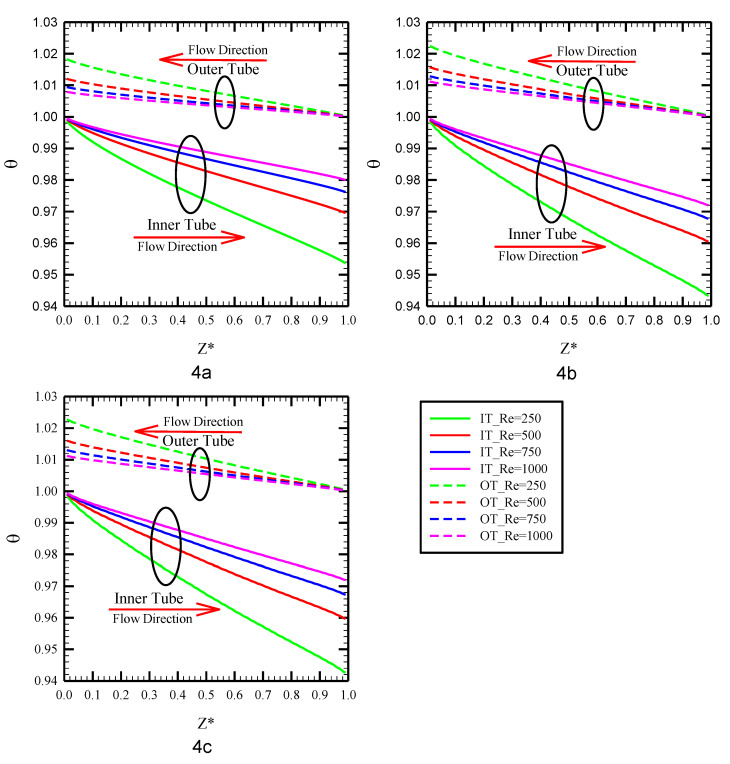
Local bulk temperature distribution along the length of the tube at Reynolds number of 250 for: (**a**) PHE; (**b**) Co-STT; and (**c**) Counter-STT.

The distribution of bulk temperature in the heat exchanger inner and outer tubes throughout the channel length for water as the working fluid at various Reynolds numbers is displayed in Figure 4. The fluid temperature from the inlet to outlet is different for inner and outer tubes so that the fluid flowing through the outer tube experiences fewer changes than that for the inner tube. The reason is because of the difference in mass flow rate values of inner and outer tubes since the higher mass flow rate at the outer tube causes fewer changes in the fluid temperature along the heat exchanger length. It is also visible that the fluid temperature undergoes more changes in lower Reynolds numbers due to the lower fluid velocity resulting in more time for fluids flowing through the inner and outer tubes from the heat exchanger’s inlet to outlet to exchange heat. Another point that is visible in this figure is that, at a given Reynolds number, the fluid outlet temperature in PHE is different compared with Co-STT and Counter-STT cases so that the twisted tape cases show a higher temperature difference between the heat exchanger inlet and outlet in comparison with the PHE. In fact, at a given Reynolds number, higher variation in fluid temperature from inlet to outlet implies higher heat transferring between the fluid flowing through inner and outer tubes resulting in higher heat exchanger efficiency. As a result, applying twisted tapes in both tubes of the heat exchanger causes a thermal enhancement. The reason is that in the twisted tape cases, secondary flow is created as a result of flow swirling, which consequently improves the flow mixing, disturbs the thermal boundary layer and enhances the heat transfer rate. In other words, the twisted tape redirects the colder core fluid with a better cooling capability to the heat exchanger interface wall causing higher thermal performance of the system.

**Figure 5:**
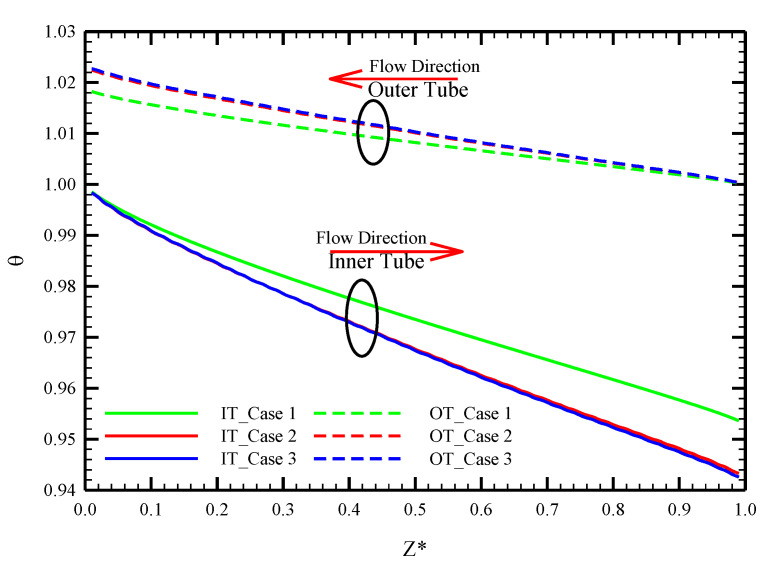
Local bulk temperature distribution along the length of the tube for PHE, Co-STT and Counter-STT at Reynolds number of 250.

For a better comparison between the bulk temperature distributions of the proposed cases along the heat exchanger length at a given Reynolds number, Figure 5 illustrates the local bulk temperature at the Re = 250. It is visible that the temperature difference of the heat exchanger’s inlet and outlet is higher in Co-STT and Counter-STT cases in comparison with the PHE. Comparing the two cases of Co-STT and Counter-STT, a slightly higher fluid temperature difference of the heat exchanger’s inlet and outlet is visible in the Counter-STT case due to the opposite angular direction of the twisted tapes in the inner and outer tubes.

**Figure 6:**
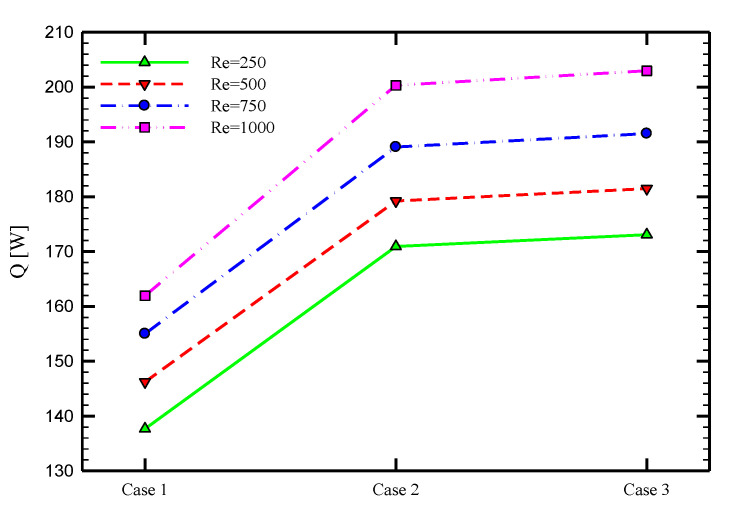
Local bulk temperature distribution along the length of the tube for PHE, Co-STT and Counter-STT at Reynolds number of 250.

Figure 6 displays the heat flux between the inner and outer tubes for different proposed cases. As shown, the transferred heats for the double-pipe inserts (Cases 2 and 3) are much higher than that for Case 1. Besides, Case 3 has a slight advantage compared with Case 2 according to the transferred heat flux between the pipes. For example, for the Reynolds number of 250 and ϕ=0, the heat flux for Case 3 is 6.67 W, which is 29.62 and 4.82% higher than Cases 1 and 2, respectively.

**Figure 7:**
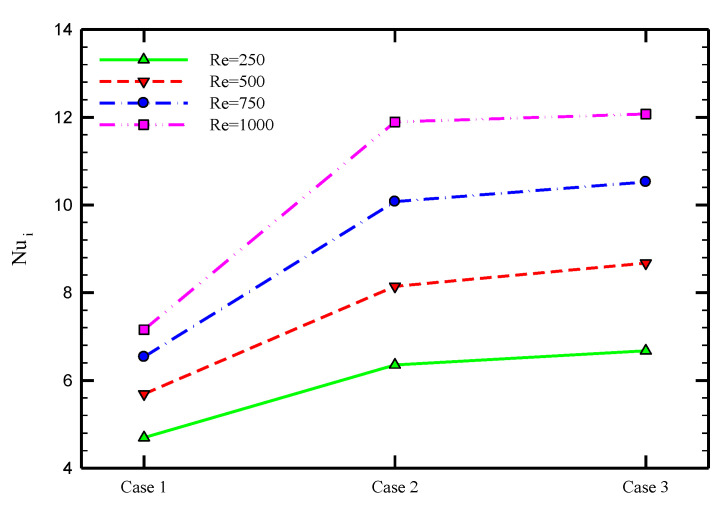
Average Nusselt number for PHE, Co-STT, and Counter-STT at various Reynolds numbers.

To better quantify the heat transfer rate, the average variations of the Nusselt number of the heat exchanger’s inner and outer tubes for various cases of PHE, Co-STT, and Counter-STT are shown in Figure 7 at four different Reynolds number. Using Co-STT and Counter-STT cases increases the average Nusselt number by about 35.24 and 42.09% at Reynolds number of 250; 43.18 and 52.46% at Reynolds number of 500; 54.15 and 61.05% at Reynolds number of 750; and 66.20 and 68.69% at Reynolds number of 1000 with respect to the PHE, respectively. Analyzing the enhanced Nusselt number percentage for different cases of the inserted twisted tape implies that the Counter-STT case leads to higher thermal performance compared with the Co-STT case, and both are significantly better in heat transfer enhancement than PHE. In addition, higher Nusselt number values for higher Reynolds numbers due to the enhancement of the advection phenomenon and more fluid momentum is visible at this figure.

**Figure 8:**
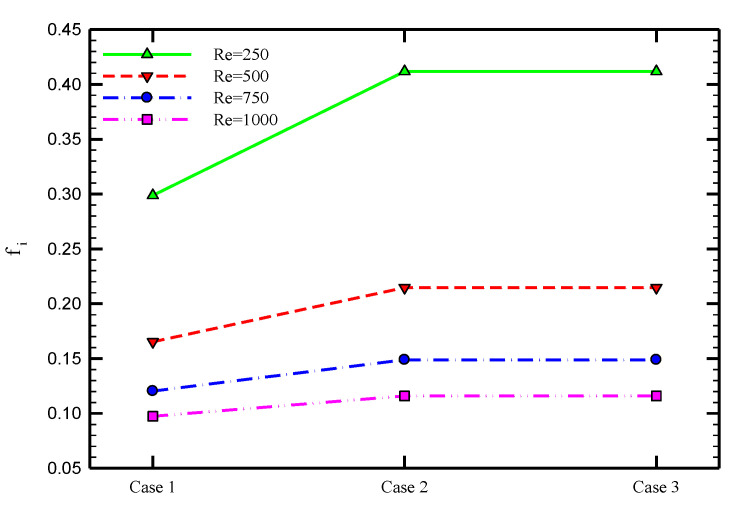
Variations of friction coefficient for different cases of PHE, Co-STT, and Counter-STT at various Reynolds numbers.

Figure 8 demonstrates the friction coefficient variations for the cases of PHE, Co-STT, and Counter-STT at different Reynolds numbers. Applying twisted tape increases the friction coefficient due to the added surface area and flow blockage created by the twisted tape inserted in the tube. Furthermore, there is no difference between the friction coefficient values of the Co-STT and Counter-STT cases as the secondary flow intensity, added surface area, and flow blockage effects are the same in both cases. Moreover, the friction coefficient decreases at higher Reynolds numbers. This can be explained using Equation (17) in which there exists velocity to the power of two in the denominator of friction coefficient correlation.

**Figure 9:**
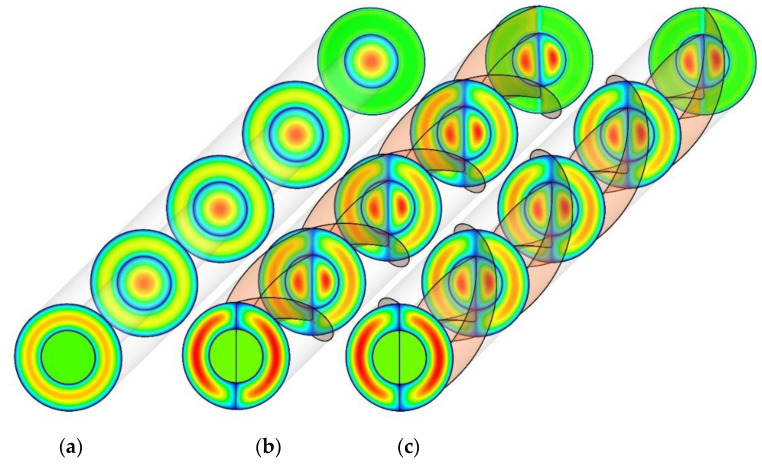
Cross-sectional velocity contours at the Reynolds number of 250 for: (**a**) PHE; (**b**) Co-STT; and (**c**) Counter-STT.

Figure 9 shows the cross-sectional velocity contours for PHE, Co-STT, and Counter-STT at Re = 250. It is visible that applying the twisted tapes at both inner and outer tubes redirects the core fluid with higher velocity and heat transfer capacity to the vicinity of the interface wall causing higher thermal performance. There is no difference between the velocity contours of Co-STT and Counter-STT cases since the direction of the twisted tape does not affect the velocity distribution. In addition, comparing the red colors of different cases, showing higher velocity at the fluid core, it can be implied that the twisted tapes create a secondary flow and better mixing causing higher velocity and advection phenomenon near the interface wall, and, as a result, a higher heat transfer rate is obtained.

**Figure 10:**
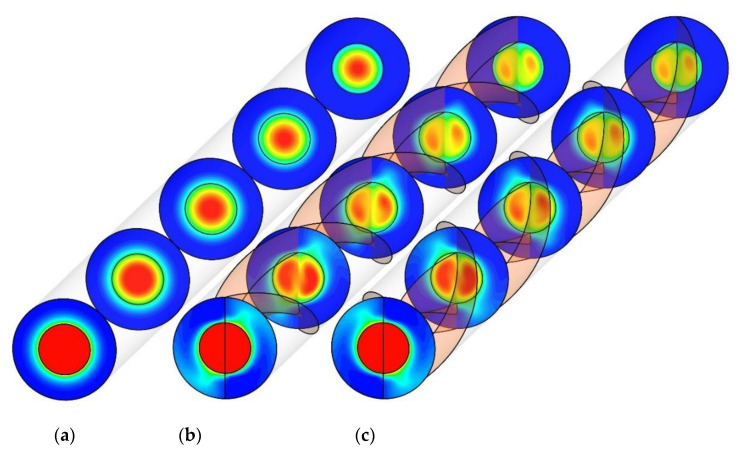
Cross-sectional temperature contours at Reynolds number of 250 for: (**a**) PHE; (**b**) Co-STT; and (**c**) Counter-STT.

Figure 10 displays the cross-sectional temperature contours for PHE, Co-STT, and Counter-STT at the Reynolds number of 250. The PHE temperature contours show the normal development of thermal boundary layer in both inner and outer tubes as the fluid flows through the unit; however, the temperature contours at the cases of Co-STT and Counter-STT are different so that the thermal boundary layer disturbance occurs and secondary flow and better flow mixing affect the boundary layer causing higher heat exchanger efficiency. Moreover, looking at the temperature distributions at the tubes’ outlet implies the fact that using twisted tape causes a higher amount of fluid to participate in the heat transfer process between the tubes due to the presence of secondary flow and better flow mixing by substituting the fluid near the interface wall with the core fluid and vice versa. Comparing the temperature distribution for the two cases of Co-STT and Counter-STT shows that, in the Counter-STT case, the secondary flow at both tubes causes the regions with thinnest thermal boundary layer in both tubes to be in direct contact with each other and are placed in the same angular direction, resulting in higher thermal performance in comparison with Co-STT case. Thus, the regions in the outer tube with the thinnest thermal boundary layer are in direct contact with the regions in the inner tube with the thickest thermal boundary layer.

**Figure 11:**
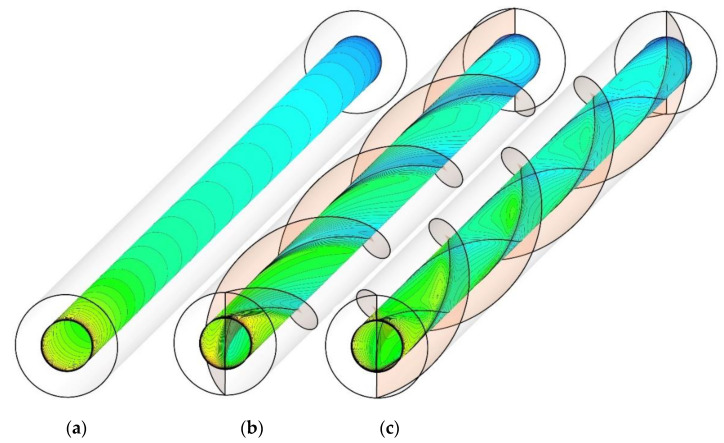
Temperature contours on the interface wall of the heat exchanger at Reynolds number of 250 for: (**a**) PHE; (**b**) Co-STT; and (**c**) Counter-STT.

Figure 11 illustrates the temperature contour at the interface wall of the heat exchanger for PHE, Co-STT, and Counter-STT at the Reynolds number of 250. The temperature distribution on the interface wall implies the intensity of the secondary flow affecting the interface wall. In the PHE, the temperature enhances uniformly as the fluid flows along the heat exchanger length showing the thermal boundary layer development. In contrast, in the presence of twisted tapes in the inner and outer tubes, the disturbance in the thermal boundary layer increases, which is enhanced in Counter-STT compared with Co-STT, implying higher thermal performance.

### 4.2. Effect of Nanoparticle Concentration on the Performance of Counter-STT Compared with Co-STT

**Figure 12:**
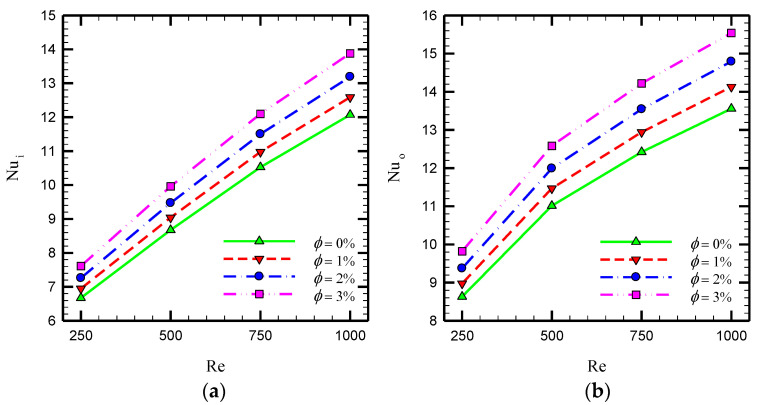
Average Nusselt number for different volume fractions of nanoparticles in terms of Reynolds number for Counter-STT case at (**a**) inner and (**b**) outer tubes of the heat exchanger.

To investigate the effects of Al_2_O_3_ nanofluid in the case of Counter-STT, Figure 12 depicts the variations of Nusselt number at the inner and outer tubes in terms of Reynolds number at various volume concentrations of the nanofluid. For all Reynolds numbers, adding nanoparticles to the base-fluid augments the Nusselt number at both inner and outer tubes of the heat exchanger results in the higher thermal efficiency of the heat exchanger. Dispersing nanoparticles modifies the fluid thermal properties, including thermal conductivity, resulting in a higher heat transfer rate.

**Figure 13:**
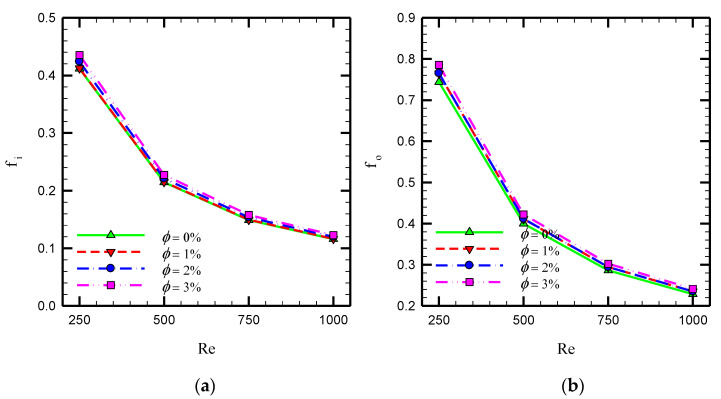
Variations of friction coefficient in terms of Reynolds number for different volume fractions of nanoparticles for Counter-STT case (**a**) inner and (**b**) outer tubes of the heat exchanger.

Figure 13 depicts the variations of friction factor at the inner and outer tubes in terms of Reynolds number at various volume concentrations of the nanofluid in the case of Counter-STT. Using nanoparticles enhances the viscosity of the fluid, causing higher friction coefficient magnitudes. Moreover, using nanofluids in such geometries, including twisted tape inserts causing intense flow mixing and secondary flow, seems to be beneficial as it prevents undesirable phenomena, including the possible agglomeration and sedimentation of nanoparticles.

**Figure 14:**
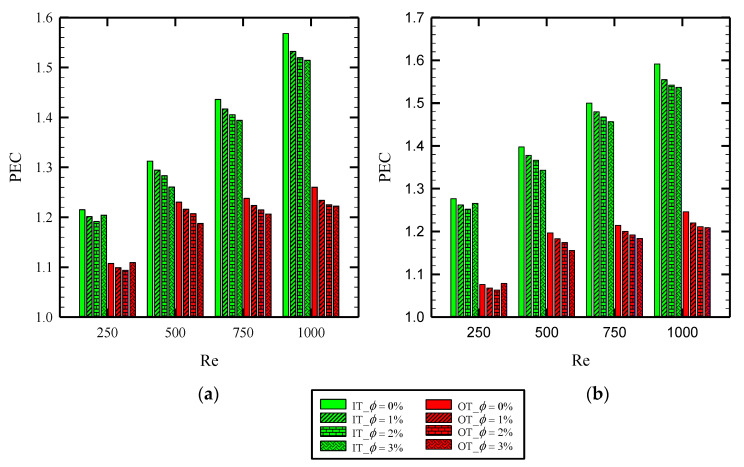
PEC parameter at different Reynolds numbers and nanoparticles volume fractions for different cases of: (**a**) Co-STT; and (**b**) Counter-STT.

As discussed above, the twisted tape inserts augment both the heat transfer rate as a desirable objective and friction coefficient as an undesirable parameter. Therefore, there is a trade-off between the benefits of using twisted tape in thermal performance enhancement and the side effects in forcing more pumping power to the system. To analyze this issue, the dimensionless PEC introduced in Equation (21) is discussed here. In fact, this number is employed to evaluate the practical use of any enhancement techniques in the viewpoint of energy-saving potential. Generally, higher values of PEC imply superior energy saving. Figure 14 illustrates the PEC parameter for PHE, Co-SST, and Counter-SST at the inner and outer tubes. Using nanofluid and increasing its volume concentration results in higher PEC values showing more enhanced heat transfer than enhanced friction coefficient in such configurations. In addition, it can be observed that, for a higher Reynolds number, the PEC number enhances as well, implying better energy saving in higher velocities of the inlet fluid flowing through both inner and outer tubes. Moreover, the figure shows that the Counter-STT model yields higher PEC values compared with that for the Co-STT model in all Reynolds numbers and nanofluid volume concentrations. Ultimately, the highest value of PEC number is equal to 1.40 and 1.26 in the inner and outer tubes, which are found at Re = 1000, the volume fraction of 3%, and Counter-STT model.

## 5. Conclusions

Benefiting from the compound heat transfer enhancement techniques of overlapped twisted tape and nanofluid, this study numerically investigated the flow of Al_2_O_3_ nanofluid through a counter flow double pipe in which both inner and outer tubes are equipped with overlapped twisted tape inserts in two models of the same (Co-STT) and opposite (Counter-STT) angular directions. A parametric study was conducted to evaluate the effect of key design variables, including the angular direction, Reynolds number, and nanofluid volume fraction on the thermo-hydraulic characteristics of such a configuration. Dimensionless number of PEC was employed to assess the trade-off between enhancement in heat transfer and pressure drop. The following outcomes are obtained from this study:

- Using Co-STT and Counter-STT, respectively, increase the average Nusselt number by about 35.2–66.2% and 42.1–68.7% over the Reynolds number ranging 250–1000 due to the creation of secondary flow and more intense flow mixing in the presence of overlapped twisted tape inserts. Ultimately, it was shown that the highest values of PEC number are equal to 1.40 and 1.26 for the inner and outer tubes, respectively, which are found at the Reynolds number of 1000, the volume fraction of 3% and Counter-STT model. The finding of this research provides a framework for researchers working on novel combined techniques for heat transfer enhancement.

## Figures and Tables

**Table 1:** Water–Al_2_O_3_ nanofluid thermo-physical properties.

**Nanoparticle Properties**				
	$\rho \left(\frac{kg}{m^{3}}\right)$	$c_{p}\left(\frac{J}{kgK}\right)$	$k\left(\frac{W}{mK}\right)$	μ(Pa s)
	3880	733	36	-
**Nanofluid Properties**				
	$\rho \left(\frac{kg}{m^{3}}\right)$	$c_{p}\left(\frac{J}{kgK}\right)$	$k\left(\frac{W}{mK}\right)$	μ(Pa s)
ϕ=0	998.2	4182	0.6	1.00 × 10^−03^
ϕ=0.01	1027.018	4147.51	0.616618	0.001089
ϕ=0.02	1055.836	4113.02	0.633833	0.001199
ϕ=0.03	1084.654	4078.53	0.651644	0.001334

**Table 2:** Grid independency analysis.

Case	Number of Elements	Nusselt Number	Error (%)
3	2,000,000	15.53	-
4	2,750,000	14.36	7.53
5	3,500,000	13.87	3.41
6	4,250,000	13.65	1.59
7	5,000,000	13.55	0.73

**Table 3:** Deviation of current numerical results from experimental data [35]. Reproduced with permission from [35]. Copyright Elsevier, 2020.

Experimental Data [35]	Present Numerical Results	Error (%)
11.6901	11.6316	0.5004
13.4444	13.31	0.9997
14.9064	14.7573	1.0002
15.462	16.0032	3.5002
16.7485	16.9997	1.4999
17.2749	18.1386	4.9998
18.6491	18.9288	1.4998
19.2047	19.7808	2.9998
20.3158	20.4174	0.5001

## Nomenclature

A	area (m^2^)	**Greek symbols**	
c_p_	specific heat transfer (J/kg.K)	ϑ	kinematic viscosity (m^2^/s)
f	friction coefficient	μ	dynamic viscosity (kg/ms)
f_0_	friction coefficient of plain channel	ρ	density (kg/m^3^)
F	body force (N)	ϕ	nanofluid concentration
g	gravitational acceleration (m/s^2^)	θ	dimensionless temperature
h	heat transfer coefficient (W/m^2^K)	**Subscripts**	
k	thermal conductivity (W/mK)	ave	average
n	number of phases	b	bulk
p	pressure (Pa)	m	mixture
q″	heat flux (w/m^2^)	f	fluid
Re	Reynolds number	i	inner tube
T	temperature (K)	o	outer tube
V	velocity vector (m/s)	nf	nanofluid
V_dr_	drift velocity	**Abbreviations**	
V_f_	fluid velocity	Co-STT	co-swirling twisted tapes
V_p_	particle velocity	Counter-STT	counter-swirling twisted tapes
		PHE	plain heat exchanger
		IT	inner tube
		OT	outer tube
